# Different Effects of Intramedullary Injection of Mesenchymal Stem Cells During the Acute vs. Chronic Inflammatory Phase on Bone Healing in the Murine Continuous Polyethylene Particle Infusion Model

**DOI:** 10.3389/fcell.2021.631063

**Published:** 2021-03-19

**Authors:** Takeshi Utsunomiya, Ning Zhang, Tzuhua Lin, Yusuke Kohno, Masaya Ueno, Masahiro Maruyama, Claire Rhee, Ejun Huang, Zhenyu Yao, Stuart B. Goodman

**Affiliations:** ^1^Department of Orthopaedic Surgery, Stanford University, Stanford, CA, United States; ^2^Department of Bioengineering, Stanford University, Stanford, CA, United States

**Keywords:** inflammation, bone healing, macrophage, mesenchymal stem cell, osteolysis

## Abstract

Chronic inflammation is a common feature in many diseases of different organ systems, including bone. However, there are few interventions to mitigate chronic inflammation and preserve host tissue. Previous *in vitro* studies demonstrated that preconditioning of mesenchymal stem cells (pMSCs) using lipopolysaccharide and tumor necrosis factor-α polarized macrophages from a pro-inflammatory to an anti-inflammatory phenotype and increased osteogenesis compared to unaltered MSCs. In the current study, we investigated the local injection of MSCs or pMSCs during the acute versus chronic inflammatory phase in a murine model of inflammation of bone: the continuous femoral intramedullary polyethylene particle infusion model. Chronic inflammation due to contaminated polyethylene particles decreased bone mineral density and increased osteoclast-like cells positively stained with leukocyte tartrate resistant acid phosphatase (TRAP) staining, and resulted in a sustained M1 pro-inflammatory macrophage phenotype and a decreased M2 anti-inflammatory phenotype. Local injection of MSCs or pMSCs during the chronic inflammatory phase reversed these findings. Conversely, immediate local injection of pMSCs during the acute inflammatory phase impaired bone healing, probably by mitigating the mandatory acute inflammatory reaction. These results suggest that the timing of interventions to facilitate bone healing by modulating inflammation is critical to the outcome. Interventions to facilitate bone healing by modulating acute inflammation should be prudently applied, as this phase of bone healing is temporally sensitive. Alternatively, local injection of MSCs or pMSCs during the chronic inflammatory phase may be a potential intervention to mitigate the adverse effects of contaminated particles on bone.

## Introduction

Osseointegration of cementless total joint arthroplasty (TJA) is similar to primary fracture healing, in which an acute-transient inflammatory reaction plays a prominent role to obtain bone healing (Loi et al., 2016a). However, wear particles and other byproducts from TJA can induce chronic inflammation and bone resorption (periprosthetic osteolysis) (Goodman, 2007). Revision TJA, due to inadequate fixation during the early phase after surgery, or particle-associated osteolysis during the chronic inflammatory phase, is technically more demanding with higher complication rates than primary TJA (Kurtz et al., 2014). Thus, new strategies for enhancing acute inflammation transiently and reducing chronic inflammation are required to facilitate bone healing and improve the survivorship of TJA.

Wear particles activate macrophages that stimulate both paracrine and autocrine cell interactions and initiate the inflammatory cascade. This triggers the differentiation, maturation and activation of osteoclasts, resulting in osteolysis (Goodman et al., 2014; Qiu et al., 2020). In addition, macrophages play an important role in the recruitment and differentiation of mesenchymal stem cells (MSCs) during bone regeneration (Pajarinen et al., 2019). Therefore, MSC-based therapy has great potential to modulate inflammatory responses and regeneration of bone and other tissues (Shi et al., 2010; Iaquinta et al., 2019).

Preconditioning of MSCs (pMSCs) using lipopolysaccharide (LPS) and tumor necrosis factor-α (TNF- α) has been shown to “license” or activate the MSCs, increase osteogenic differentiation, and polarize macrophages from an M1 pro-inflammatory phenotype to an M2 anti-inflammatory phenotype *in vitro* (Lin et al., 2019). However, due to the different inflammatory profiles of the early and late phases of bone healing, the effects of pMSCs on acute versus chronic inflammation are unknown. We hypothesized that by using local delivery of pMSCs, (1) bone healing during the acute inflammatory phase could be facilitated, and (2) osteolysis due to chronic inflammation induced by polyethylene particles could be suppressed in the murine continuous polyethylene particle infusion model. In the current study, we tested these hypotheses using our established *in vivo* model.

## Materials and Methods

### Animals

The experimental design was approved by the Institutional Administration Panel for Laboratory Animal Care at Stanford University (Protocol number: 17566), and Institutional Guidelines for the Care and Use of Laboratory Animals were observed in all aspects of this project. We used 10–12-week-old BALB/c male mice (Jackson Laboratory, Bar Harbor, ME). All the animals were kept on a 12-h light-and-dark cycle and fed a standard diet with food and water *ad libitum*.

### Isolation of Murine MSCs

Bone marrow derived MSCs were isolated as previously described (Lin et al., 2017, 2018, 2019). Briefly, bone marrow was collected from the femurs and tibias of 10-week-old BALB/c male mice, then using a 25-gauge needle, the cells were carefully suspended and passed through a 70-μm strainer into a centrifuge tube. After spinning down at 400g for 5 minutes, supernatant was removed, and cells were resuspended in alpha-minimal essential medium (α-MEM, Thermo Fisher Scientific, Waltham, MA, United States) supplied with 10% MSC certified with fetal bovine serum (FBS, Invitrogen, Carlsbad, CA, United States) and antibiotic antimycotic solution (100 units of penicillin, 100 μg of streptomycin 0.25 μg of Amphotericin B per mL; Hyclone, Thermo Scientific). The cells plated on the T-175 flasks were incubated overnight in 37 degrees at 5% CO2. The 30 ml fresh medium was replaced the next day to remove the unattached cells and the cells were designated as passage 1. The culture media was changed twice a week, and the cells were allowed to grow to confluence around 3–4 weeks later. The cells were washed with 10 ml PBS, detached with 5 ml trypsin, and incubated for 2 min in 37 degrees at 5% CO2. The detached cells were flushed with 15 ml media into a centrifuge tube and spun down at 400g for 5 minutes. Cells were resuspended and plated at a density of 4,000 cells/cm^2^. This procedure for subculture was repeated twice until pure MSCs were isolated using the murine mesenchymal stem cell enrichment kit (STEMCELL Technologies, Vancouver, Canada) in passage 4. The cellular morphology was observed under a microscope (Axio Observer 3.1, Zeiss, Oberkochen, Germany). At passage 4, the immunophenotype of the isolated MSCs (CD105+/CD73+/CD90.2+/CD44+/Sca1+/CD45−/ CD34−/CD11b−) as defined by the International Society for Cell Therapy (Dominici et al., 2006) was characterized by LSR II flow cytometer (BD Bioscience).

### Preconditioning of MSCs

**FIGURE 1:**
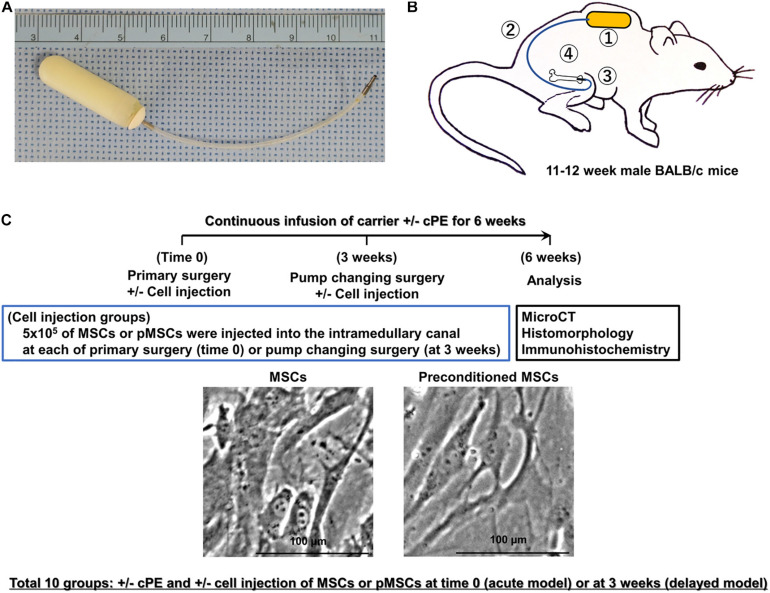
Implants, animal model, treatment groups, and timeline. **(A)** Implants used in this murine model include a mini-osmotic pump containing contaminated polyethylene particles (cPE) with 10 ng/mL of lipopolysaccharide (LPS) or carrier, 6 cm of vinyl catheter and hollow titanium rod (6 mm long, 23 gauge). **(B)** Schematic drawing of this murine model with a mini-osmotic pump containing cPE or carrier. The mini-osmotic pump with cPE or carrier alone is implanted in the subcutaneous tissue at the dorsum of the mouse (①). The pump is then connected via a subcutaneous vinyl catheter (②) to a hollow titanium rod (③) that is press fit into the intramedullary canal of the right distal femur (④). **(C)** Groups and timeline. There were ten groups with/without cPE and with/without injection of 5 × 10^5^ mesenchymal stem cells (MSCs) or preconditioned MSCs (pMSCs) into the intramedullary space of the femur through a hollow titanium rod by a Hamilton syringe at the time of primary surgery (time 0) or 3 weeks after primary surgery as follows: (1) cPE (−) control, (2) cPE (+) treatment, (3) cPE (−) MSCs (time 0), (4) cPE (−) pMSCs (time 0), (5) cPE (+) MSCs (time 0), (6) cPE (+) pMSCs (time 0), (7) cPE (−) MSCs (3 weeks), (8) cPE (−) pMSCs (3 weeks), (9) cPE (+) MSCs (3 weeks), and (10) cPE (+) pMSCs (3 weeks). Each group included 7 mice. Pumps were also replaced with new ones containing the same infusion as the first 3 weeks for the following three weeks in every group. Immunohistochemistry, histopathological analysis and MicroCT analysis were carried out 6 weeks after primary surgery. The carrier for the particles was 10%BSA/PBS; cPE, Contaminated polyethylene particles with lipopolysaccharide.

According to our established protocol for preparation of pMSCs (Lin et al., 2017, 2019), murine MSCs were seeded at a density of 5,000 cells/cm^2^, and treated with a combination of 20 ng/ml tumor necrosis factor alpha (TNF-α) and 20 ng/ml lipopolysaccharide (LPS, Sigma-Aldrich, St Louis, MO, United States) for 3 days. At day 4, the cells were washed three times with Phosphate-Buffered Saline (PBS), and replaced with fresh media containing 40 μg/ml Polymyxin B1 to inactivate the any remaining LPS. For the treatment groups, 5 × 10^5^ murine primary MSCs or 5 × 10^5^ pMSCs in 10 μl of PBS were injected into the intramedullary canal through the hollow titanium rod by a Hamilton syringe during primary surgery (time 0) or pump changing surgery (at 3 weeks), at which time the pumps were replaced with new ones for the following three weeks (Figure 1).

### Ultra-High Molecular Weight Polyethylene Particles and Preparation of Osmotic Pumps

Polyethylene particles were prepared as previously described (Lin et al., 2017, 2019). Briefly, Ceridust 3610 polyethylene particles (Clariant Corporation, CA, United States) were washed and resuspended in ethanol then filtered through a 20 μm pore membrane to exclude larger particles. Finally, the filtered particle size was measured as 4.62 ± 3.76 μm using a scanning electron microscope (Zeiss Sigma FESEM, Zeiss Sigma, CA, United States) in the Cell Sciences Imaging Facility at Stanford University.

The particles were dried for 3 days and resuspended in PBS containing 5% Bovine Serum Albumin (BSA, Thermo Fisher Scientific) at a concentration of approximately 3.1 × 10^10^ particles/ml. The sterility of the particles was confirmed by the endpoint chromogenic Limulus Amebocyte Lysate assay (Lonza, Portsmouth, NH). Contamination of the polyethylene particles was accomplished by exposing them to 10 ng/mL of LPS (Sigma-Aldrich St Louis, MO) (Greenfield et al., 2010). The carrier, 10% BSA/PBS, with/without contaminated polyethylene particles (cPE, working concentration: 15 mg/ml for the *in vivo* experiments) was then filled into an Alzet mini-osmotic pump (Model 2006, DURECT corporation, Cupertino, CA, United States). The pump was connected with a vinyl catheter and a hollow titanium rod. Finally, all the pumps with vinyl catheters and hollow titanium rods were placed in the incubator (37°C) for 3 days before the surgeries. The cPE were infused for total 6 weeks with a mean pumping rate of 0.15 ml/hour; thus, the total amount of cPE that was infused per mouse for 6 weeks was 4.7 × 10^9^ particles theoretically.

### Mouse Model of Particle-Induced Osteolysis and Experimental Design

We used 11–12-week-old BALB/c male mice, and the continuous femoral intramedullary polyethylene particle infusion model as previously described (Figure 1A; Ren et al., 2011; Lin et al., 2016; Sato et al., 2016; Nabeshima et al., 2017). Briefly, under preoperative analgesia (0.1 mg/kg of buprenorphine, subcutaneously) and inhalation anesthesia with 2% isoflurane in 100% oxygen at a flow of 1L/min on a warm small animal surgery station, we approached the right knee joint via a lateral parapatellar incision, and pierced through the intercondylar notch into the medullary cavity using a series of needles (25, 23, and 22 gauges) sequentially. A hollow titanium rod (6 mm long, 23 gauge) was then press-fit into the distal canal of the femur through the intercondylar region. The Alzet mini-osmotic pumps containing 10% BSA/PBS with/without cPE were implanted subcutaneously and dorsally through a second incision around the right shoulder of the mouse; the pump was connected to the implanted rod via subcutaneous vinyl catheter tubing. Five hundred thousand of the MSCs or pMSCs in 10 μl of PBS were injected by a Hamilton syringe into the intramedullary space of the femur via the implanted hollow titanium rod at either the primary surgery (acute model) or 3 weeks after the primary surgery (delayed model). Three weeks after the primary surgery, pumps were changed into new ones containing 10% BSA/PBS with/without cPE, which were infused for the following three weeks (Figures 1B,C). Skin incisions were closed with 5-0 Ethilon sutures. Each mouse was able to access food and water freely in their own cage.

Thus, there were 10 groups with/without cPE and with/without MSCs or pMSCs injected into the intramedullary space of the femur at the primary surgery or at 3 weeks after surgery as follows: (1) cPE (−) control, (2) cPE (+) treatment, (3) cPE (−) MSCs (time 0), (4) cPE (−) pMSCs (time 0), (5) cPE (+) MSCs (time 0), (6) cPE (+) pMSCs (time 0), (7) cPE (−) MSCs (3 weeks), (8) cPE (−) pMSCs (3 weeks), (9) cPE (+) MSCs (3 weeks), and (10) cPE (+) pMSCs (3 weeks) (Figure 1C, and Table 1). For the current *in vivo* experiment, power analysis was carried out to determine a sufficient number of mice based on detecting a significant difference of 1.3 standard deviation (effect size of 1.3) with a power of 90% (alpha = 0.05, beta = 0.10). Changes in bone morphometric parameters were based on the results of histomorphometry and MicroCT from our previous studies. Power analysis concluded that an n = 7 animals were necessary for this *in vivo* experiment. Each group therefore included 7 mice. Mice were euthanized 6 weeks after the primary surgery and the titanium rod was removed from the distal femur before histopathological analysis and MicroCT scanning.

**TABLE 1:** Experimental groups of the murine model in the present study.

Group	Infusion of carriers	Infusion of cPE	Injection of MSCs	Injection of pMSCs
cPE (−) control	✓			
cPE (−) MSCs (time 0)	✓		at time 0	at time 0
cPE (−) pMSCs (time 0)	✓		at time 0	at time 0
cPE (−) MSCs (3 weeks)	✓		at 3 weeks	at 3 weeks
cPE (−) pMSCs (3 weeks)	✓		at 3 weeks	at 3 weeks
cPE (+) treatment	✓	✓		
cPE (+) MSCs (time 0)	✓	✓	at time 0	at time 0
cPE (+) pMSCs (time 0)	✓	✓	at time 0	at time 0
cPE (+) MSCs (3 weeks)	✓	✓	at 3 weeks	at 3 weeks
cPE (+) pMSCs (3 weeks)	✓	✓	at 3 weeks	at 3 weeks

*Carriers, 10% Bovine Serum Albumin (BSA)/Phosphate-Buffered Saline (PBS); cPE, contaminated polyethylene particles with lipopolysaccharide (LPS); MSCs, mesenchymal stem cells; pMSCs, preconditioned mesenchymal stem cells. Each group included 7 mice.*

### Tissue Processing for Frozen Specimens

Mice were euthanized with exposure to CO_2_ followed by cervical dislocation, and all the implants were detached before MicroCT scanning 6 weeks after the primary surgery. Femurs of operated sides were then removed, and femurs were fixed in 4% paraformaldehyde overnight and decalcified in 0.5 M ethylenediamine-tetra-acetic acid (EDTA, pH 7.4) for 2 weeks. The specimens were then placed in embedding medium (optimal cutting temperature or OCT) (Tissue Plus^®^, Fisher HealthCare, Hampton, NH) for frozen specimens. Finally, the region of interest located 3 mm from the distal end of femur was cut into transverse sections with 10 μm-thickness for subsequent staining (Lin et al., 2016).

### Immunohistochemistry for Macrophage Phenotype

For the detection of macrophages, frozen sections were exposed to 5% BSA as blocking buffer for 30 minutes at room temperature followed by incubation with the primary and secondary antibodies for 1 hour each. Macrophages were identified by immunofluorescence staining with rat anti-mouse F4/80 monoclonal antibody (CI: A3-1, Bio-Rad) followed by Alexa Fluor^®^ 594 conjugated goat anti-rat IgG (Invitrogen, Carlsbad, CA, United States). To identify the M1 pro-inflammatory macrophage phenotype, sections were also stained with rabbit anti-mouse inducible nitric oxide synthase (iNOS) polyclonal antibody (Abcum, Cambridge, United Kingdom) followed by Alexa Fluor^®^ 488 conjugated goat anti-rabbit IgG (Invitrogen, Carlsbad, CA, United States) (Ueno et al., 2020). The slides were then mounted with ProLong Gold Antifade Mount with DAPI (Life Technologies, Grand Island, NY) and the photomicrographs were observed under a fluorescence microscope (Digital Microscope, Keyence, IL). The number of cells positively stained with F4/80 and the number of cells double positively stained with both F4/80 and iNOS (the M1 pro-inflammatory macrophage phenotype) were manually counted in 3 randomly selected views of each specimen with x200 magnification by ImageJ software (National Institutes of Health, Bethesda, MA, United States). Finally, the proportion of M1 pro-inflammatory macrophages was quantified according to the following calculation: the number of cells double positively stained with both F4/80 and iNOS divided by the sum of the number of cells stained with F4/80 and the number of cells double positively stained with both F4/80 and iNOS.

To identify the M2 anti-inflammatory macrophage phenotype, different sections were also exposed to 5% BSA as blocking buffer for 30 minutes at room temperature followed by incubation with each of the primary and secondary antibodies for 1 hour each. Macrophages were identified by immunofluorescence staining with rat anti-mouse F4/80 monoclonal antibody (CI: A3-1, Bio-Rad) followed by Alexa Fluor^®^ 594 conjugated goat anti-rat IgG (Invitrogen, Carlsbad, CA, United States). Sections were stained with rabbit anti-mouse liver Arginase (Arg1) polyclonal antibody (Abcum, Cambridge, United Kingdom) followed by Alexa Fluor^®^ 488 conjugated goat anti-rabbit IgG (Invitrogen, Carlsbad, CA, United States) to identify the M2 anti-inflammatory macrophage phenotype (Ueno et al., 2020). The slides were then mounted with ProLong Gold Antifade Mount with DAPI (Life Technologies, Grand Island, NY, United States) and imaged under a fluorescence microscope (Digital Microscope, Keyence, IL). The number of cells positively stained with F4/80 and the number of cells double positively stained with both F4/80 and Arg1 (the M2 anti-inflammatory macrophage phenotype) were manually counted in 3 randomly selected views of each specimen with x200 magnification by ImageJ software. Finally, the proportion of M2 anti-inflammatory macrophages according to the following calculation: the number cells double positively stained with both F4/80 and Arg1 divided by the sum of the number of cells positively stained with F4/80 and the number of cells double positively stained with both F4/80 and Arg1.

### Detection and Measurement of Osteoclast-Like Cells and Osteoblast-Like Cells

Osteoclast-like cells and osteoblast-like cells were determined as previously described (Miron et al., 2016; Miron and Bosshardt, 2018; Brooks et al., 2019). Briefly, in the current study, TRAP staining kit (Sigma Aldrich, St. Louis, MO) was used to identify osteoclast-like cells with multi-nuclei brown staining, and were located on the bone perimeter within resorption lacunae (Sato et al., 2015; Lin et al., 2016; Nabeshima et al., 2017). Using 3 randomly selected views with x200 magnification in each specimen, in a blind manner, two investigators manually counted the number of TRAP staining positive cells, which were finally normalized by the bone area measured using ImageJ software (Nabeshima et al., 2017).

In different sections, osteoblast-like cells (Sato et al., 2015; Lin et al., 2016; Nabeshima et al., 2017) were identified using anti-alkaline phosphatase (ALP) antibody (1-Step^TM^ NBT/BCIP Substrate Solution, Thermo Scientific, Rockford, IL). Using the entire image of each specimen with x100 magnification, the percentage of brown, positively stained area for ALP was quantified based on the entire area of each section measured using ImageJ software (Ueno et al., 2020). The color threshold of ALP staining positive area was determined by consensus of three of the investigators.

### Micro-Computational Tomography

MicroCT scans were performed using eXplore Locus RS150 microCT (GE Healthcare, Fairfield, CT) with 49 μm resolution at 6 weeks after primary surgery. A three-dimensional (3D) region of interest (ROI, 4 mm × 4 mm × 3 mm) was created within the distal femur which began 3 mm from the distal end of the femur and proceeded proximally (Lin et al., 2016; Nabeshima et al., 2017). The threshold bone mineral density (BMD, mg/mm^3^) was quantified by using GEMS MicroView software (threshold: 700 HU).

The statistical analysis was conducted using Prism 7 (GraphPad Software, San Diego, CA). One-way ANOVA with Bonferroni’s *post hoc* testing was performed to compare selected groups to provide meaningful comparisons of the data for the results of the immunohistochemistry staining, TRAP staining, ALP staining and Micro CT scanning. Data were expressed as mean ± standard deviation of the mean. The difference was considered significant when the *P*-value was < 0.05.

## Results

### Contaminated Polyethylene Particles Increased the Total Number of Macrophages and the Proportion of M1 Pro-inflammatory Macrophages; MSC-Based Therapy for Both Early and Delayed Time Periods Mitigated the Effects of Contaminated Polyethylene Particles

**FIGURE 2:**
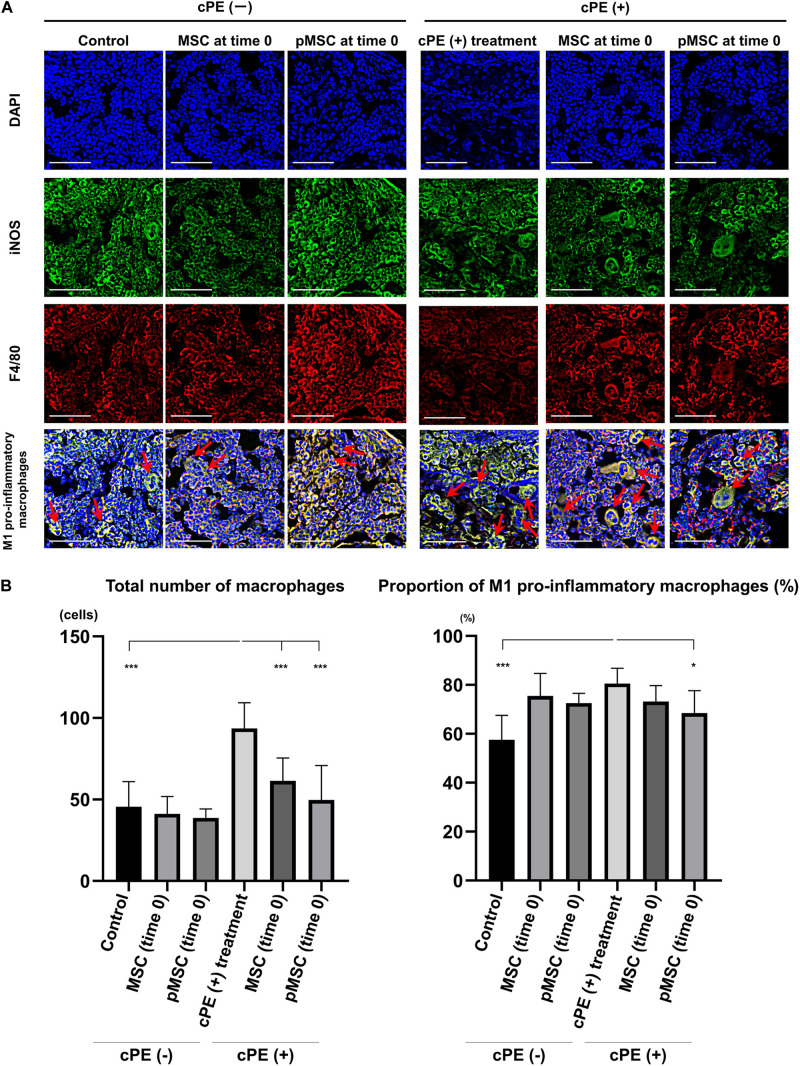
Immunohistochemistry for M1 pro-inflammatory macrophages after cell injection at the primary surgery. Representative photomicrographs of immunohistochemistry for M1 pro-inflammatory macrophages **(A)** and quantitative data **(B)** after cell injection at the primary surgery are shown. The M1 pro-inflammatory macrophages (red arrow) were identified by double positively immunofluorescence staining with both anti-F4/80 antibody (red) and anti-inducible nitric oxide synthase (iNOS) antibody (green) **(A)**. Nuclei were visualized with DAPI (blue). The proportion of M1 pro-inflammatory macrophages was quantified according to the following calculation: the number of cells double positively stained with both F4/80 and iNOS divided by the sum of the number of cells stained with F4/80 and the number of cells double positively stained with both F4/80 and iNOS. Scale bar: 50 μm, *p < 0.05 and ***p < 0.0001.

**FIGURE 3:**
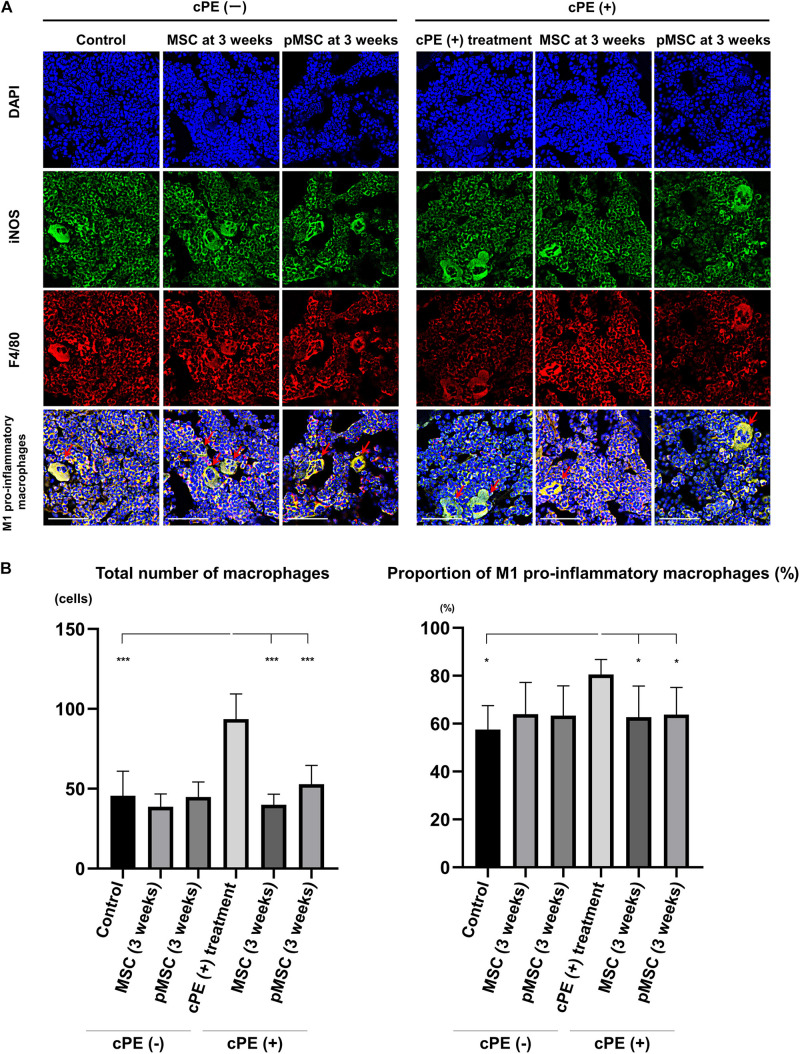
Immunohistochemistry for M1 pro-inflammatory macrophages after cell injection at the pump changing surgery 3 weeks after the primary surgery. Representative photomicrographs of immunohistochemistry for M1 pro-inflammatory macrophages **(A)** and quantitative data **(B)** after cell injection at the pump changing surgery 3 weeks after the primary surgery are shown. The M1 pro-inflammatory macrophages (red arrow) were identified by double positively immunofluorescence staining with both anti-F4/80 antibody (red) and anti-inducible nitric oxide synthase (iNOS) antibody (green). Nuclei were visualized with DAPI (blue). The proportion of M1 pro-inflammatory macrophages was quantified according to the following calculation: the number of cells double positively stained with both F4/80 and iNOS divided by the sum of the number of cells stained with F4/80 and the number of cells double positively stained with both F4/80 and iNOS. Scale bar: 50 μm, *p < 0.05 and ***p < 0.0001.

The total number of macrophages in the cPE (+) treatment group was significantly increased compared with other groups including: cPE (−) control, cPE (+) MSCs (time 0), cPE (+) pMSCs (time 0), cPE (+) MSCs (3 weeks), and cPE (+) pMSCs (3 weeks) (*P* < 0.0001). The proportion of M1 pro-inflammatory macrophages in the cPE (+) treatment group was significantly increased compared with the cPE (−) control group (*P* < 0.0001). There was no significant difference in the proportion of M1 pro-inflammatory macrophages in the cPE (−) MSCs group compared with cPE (−) pMSCs group for both early and delayed time periods. The delayed injection of MSCs and both the early or delayed injection of pMSCs significantly mitigated the effects of cPE on the proportion of M1 pro-inflammatory macrophages. There was no significant difference in the proportion of M1 pro-inflammatory macrophages in the cPE (+) pMSCs group compared with the cPE (+) MSCs group for both early and delayed time periods (Figures 2, 3).

### Contaminated Polyethylene Particles Decreased the Proportion of M2 Anti-inflammatory Macrophages; MSC-Based Therapy for Both Early and Delayed Time Periods Reversed the Effects of cPE

**FIGURE 4:**
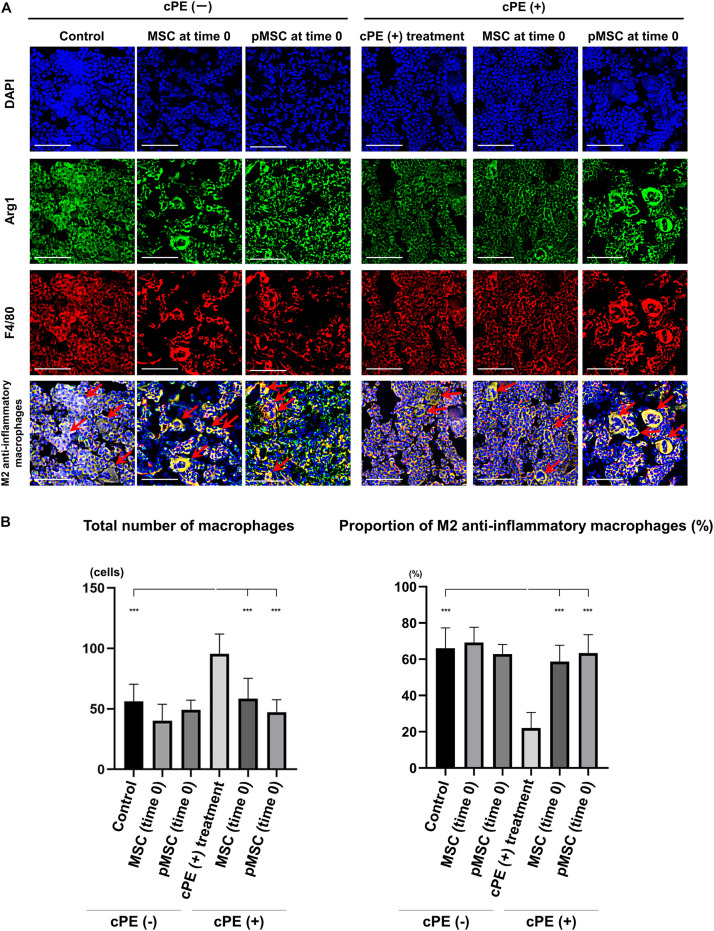
Immunohistochemistry for M2 anti-inflammatory macrophages after cell injection at the primary surgery. Representative photomicrographs of immunohistochemistry for M2 anti-inflammatory macrophages **(A)** and quantitative data **(B)** after cell injection at the primary surgery are shown. The M2 anti-inflammatory macrophages (red arrow) were identified by double positively immunofluorescence staining with both anti-F4/80 antibody (red) and anti-liver Arginase (Arg1) polyclonal antibody (green) **(A)**. Nuclei were visualized with DAPI (blue). The proportion of M2 anti-inflammatory macrophages was quantified according to the following calculation: the number cells double positively stained with both F4/80 and Arg1 divided by the sum of the number of cells positively stained with F4/80 and the number of cells double positively stained with both F4/80 and Arg1. Scale bar: 50 μm, ***p < 0.0001.

**FIGURE 5:**
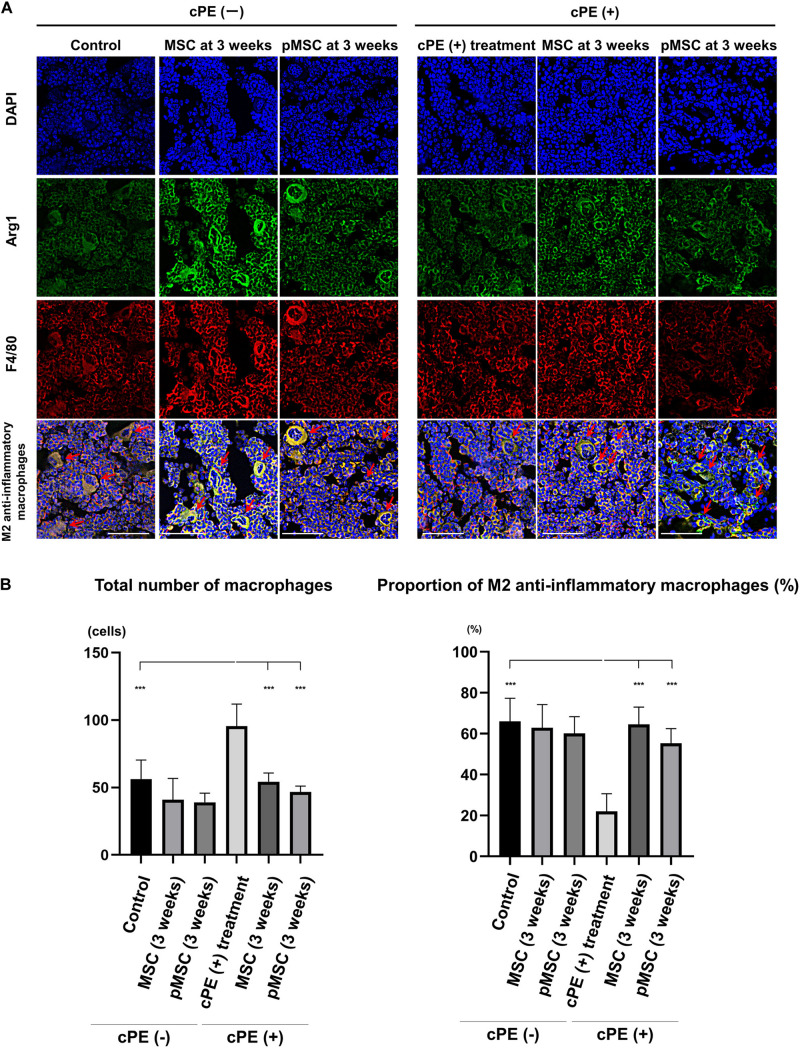
Immunohistochemistry for M2 anti-inflammatory macrophages after cell injection at the pump changing surgery 3 weeks after the primary surgery. Representative photomicrographs of immunohistochemistry for M2 anti-inflammatory macrophages **(A)** and quantitative data **(B)** after cell injection at the pump changing surgery 3 weeks after the primary surgery. The M2 anti-inflammatory macrophages (red arrow) were identified by double positively immunofluorescence staining with both anti-F4/80 antibody (red) and anti-liver Arginase (Arg1) polyclonal antibody (green) **(A)**. Nuclei were visualized with DAPI (blue). The proportion of M2 anti-inflammatory macrophages was quantified according to the following calculation: the number cells double positively stained with both F4/80 and Arg1 divided by the sum of the number of cells positively stained with F4/80 and the number of cells double positively stained with both F4/80 and Arg1. Scale bar: 50 μm, ***p < 0.0001.

In the different sections, the total number of macrophages in cPE (+) treatment group was significantly increased compared with other groups including: cPE (−) control, cPE (+) MSCs (time 0), cPE (+) pMSCs (time 0), cPE (+) MSCs (3 weeks), and cPE (+) pMSCs (3 weeks) (*P* < 0.0001). The proportion of M2 anti-inflammatory macrophages in the cPE (+) treatment group was significantly decreased compared with the cPE (−) control group (*P* < 0.0001). There was no significant difference in the proportion of M2 anti-inflammatory macrophages in the cPE (−) pMSCs group compared with the cPE (−) MSCs group for both early and delayed time periods. Both the early and delayed injection of MSCs and pMSCs reversed the effect of cPE on the proportion of M2 anti-inflammatory macrophages. There was no significant difference in the proportion of M2 anti-inflammatory macrophages in the cPE (+) pMSCs group compared with the cPE (+) MSCs group for both early and delayed time periods (Figures 4, 5).

### MSC-Based Therapy for the Delayed Time Period Decreased the Number of Cells Staining Positively With TRAP and Increased the ALP Positive Area Induced by cPE

**FIGURE 6:**
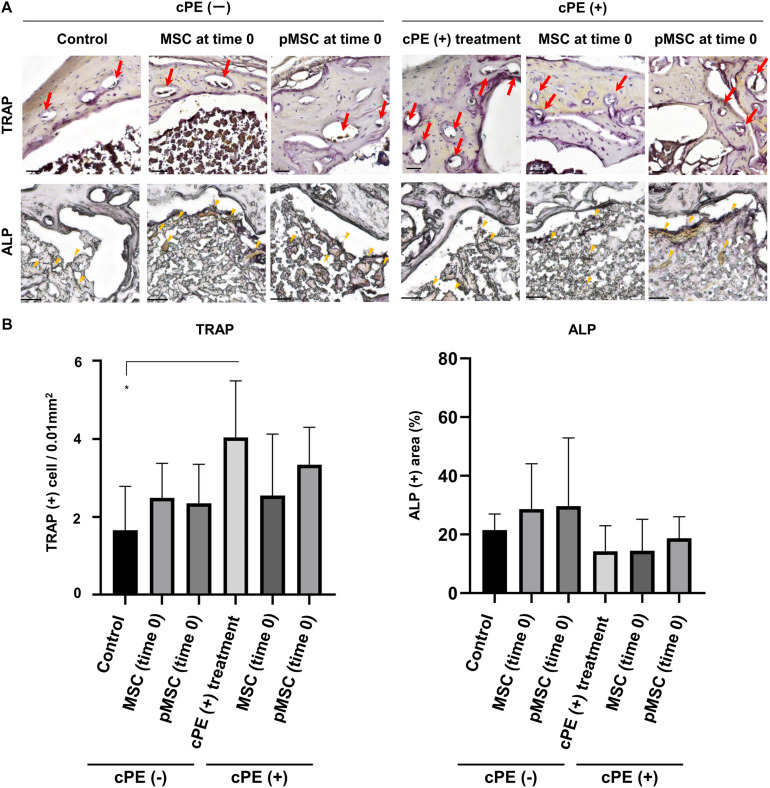
TRAP staining and ALP staining after cell injection at the primary surgery. Representative photomicrographs of leukocyte tartrate resistant acid phosphatase (TRAP) and alkaline phosphatase (ALP) staining **(A)** and quantitative data **(B)** after cell injection at the primary surgery are shown. Osteoclast-like cells with multi-nuclei, which were located on the bone perimeter within resorption lacunae were stained brown (red arrow) using TRAP staining in the upper photomicrographs **(A)**. Using 3 randomly selected views with ×200 magnification in each specimen, in a blind manner, two investigators manually counted the number of TRAP staining positive cells, which were finally normalized by the bone area measured using ImageJ software. ALP staining for osteoblast-like cells show as brown-stained cells (yellow arrowhead) in the lower photomicrographs **(A)**. Using the entire image of each specimen with ×100 magnification, the percentage of brown, positively stained area for ALP was quantified based on the entire area of each section measured using ImageJ software. The color threshold of each parameter was determined by consensus of three of the investigators Scale bar: 50 μm, *p < 0.05.

**FIGURE 7:**
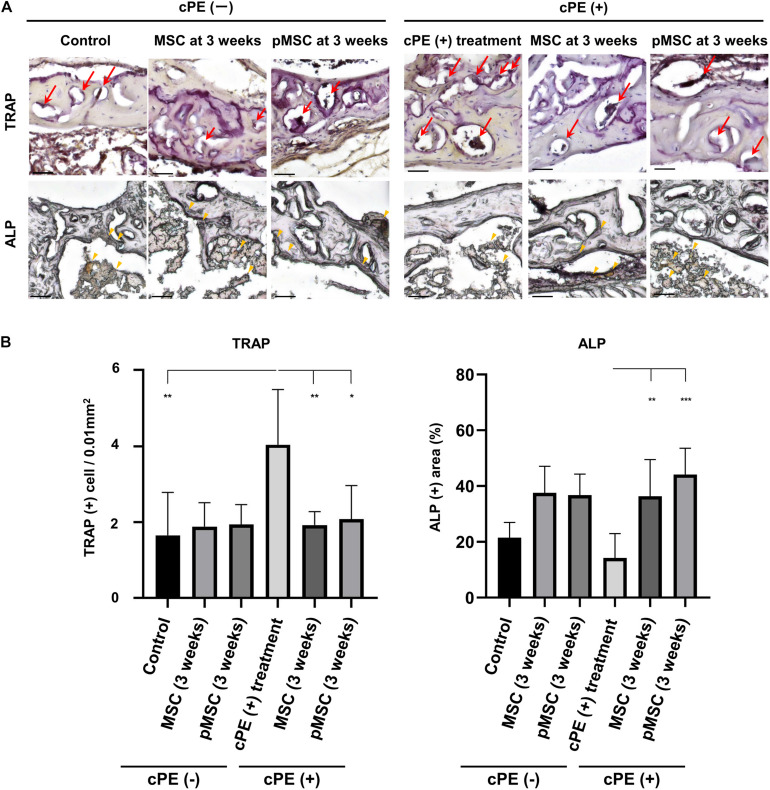
TRAP staining and ALP staining after cell injection at the pump changing surgery 3 weeks after the primary surgery. Representative photomicrographs of TRAP and ALP staining **(A)** and quantitative data **(B)** after cell injection at the pump changing surgery 3 weeks after the primary surgery are shown. Osteoclast-like cells with multi-nuclei, which were located on the bone perimeter within resorption lacunae were stained brown (red arrow) using TRAP staining in the upper photomicrographs **(A)**. Using 3 randomly selected views with ×200 magnification in each specimen, in a blind manner, two investigators manually counted the number of TRAP staining positive cells, which were finally normalized by the bone area measured using ImageJ software. ALP staining for osteoblast-like cells show as brown-stained cells (yellow arrowhead) in the lower photomicrographs **(A)**. Using the entire image of each specimen with ×100 magnification, the percentage of brown, positively stained area for ALP was quantified based on the entire area of each section measured using ImageJ software. The color threshold of each parameter was determined by consensus of three of the investigators Scale bar: 50 μm, *p < 0.05, **p < 0.001, and ***p < 0.0001 compared to the corresponding treatment group with cPE.

The number of cells staining positively with TRAP in the cPE (+) treatment group was significantly increased compared with the cPE (−) control group (*P* = 0.01). The number of cells staining positively with TRAP in the cPE (+) pMSC (time 0) group was decreased compared with the cPE (+) treatment group, but this did not reach statistical significance (Figure 6). There was no significant difference for the reduction of the number of cells staining positively with TRAP in the cPE (−) pMSCs group compared with the cPE (−) MSCs group for both early and delayed time periods. However, the number of cells staining positively with TRAP in both the cPE (+) MSCs (3 weeks) group and the cPE (+) pMSCs (3 weeks) group was significantly decreased compared with cPE (+) treatment group (*P* = 0.0004, *P* = 0.0014, respectively) (Figure 7). There was no significant difference for the reduction of the number of cells staining positively with TRAP in the cPE (+) pMSCs (3 weeks) group compared with the cPE (+) MSCs (3 weeks) group (Figure 7).

The ALP positive area in the cPE (+) treatment group was decreased compared with the cPE (−) control group, but this did not reach statistical significance. There was no significant difference in the ALP positive area in the cPE (−) MSCs group and the cPE (−) pMSCs group for both early and delayed time periods. The ALP positive area in both the cPE (+) MSCs (3 weeks) group and the cPE (+) pMSCs (3 weeks) group was significantly increased compared with the cPE (+) treatment group (*P* = 0.0008, *P* < 0.0001, respectively). Early injection of pMSCs had no effect on the ALP positive area compared with the cPE (+) treatment group (Figure 6). There was no significant difference in the ALP positive area in the cPE (+) pMSCs (3 weeks) group compared with the cPE (+) MSCs (3 weeks) group (Figure 7).

### Contaminated Polyethylene Particles Decreased Bone Mineral Density; Injection of pMSCs at the Primary Surgery Decreased Bone Mineral Density Further

**FIGURE 8:**
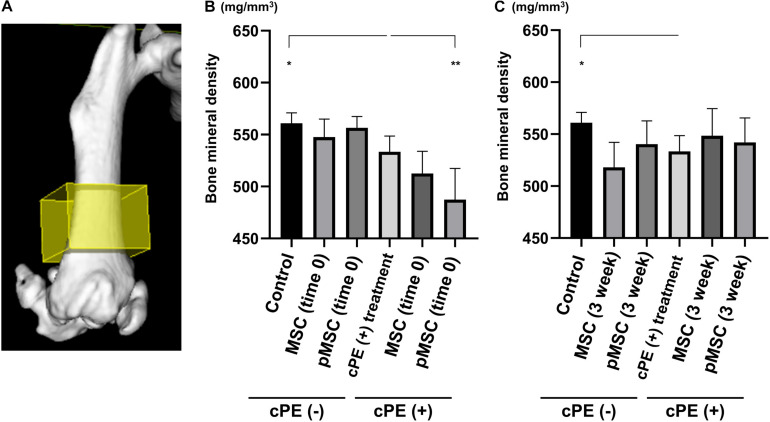
Contaminated polyethylene particles decreased bone mineral density; injection of pMSCs at the primary surgery further decreased bone mineral density. MicroCT was performed 6 weeks after primary surgery. The 3D region of interest (yellow box) was defined as 4 mm × 4 mm × 3 mm within the distal femur and began 3 mm from the distal end of the femur and proceeded proximally **(A)**. Quantitative assessments of bone mineral density are shown in each timing of cell injection at the primary surgery (time 0) and at the pump changing surgery 3 weeks after the primary surgery **(B,C)**. *p < 0.05, **p < 0.001.

BMD in the cPE (+) treatment group (530.1 ± 12.8 mg/mm^3^) was significantly decreased compared with cPE (−) control group (563.4 ± 7.7 mg/mm^3^, *P* = 0.0169). There was no significant difference in the BMD in the cPE (−) MSCs group and the cPE (−) pMSCs group for both early and delayed time periods. Unexpectedly, BMD in the cPE (+) pMSCs (time 0) group (485.5 ± 25.1 mg/mm^3^) was significantly decreased compared with the cPE (+) treatment group (*P* = 0.0005), probably by mitigating the mandatory acute inflammatory reaction. This could also be enhanced by synergism of the pro-inflammatory/osteoblastic effect of both mechanical drilling and intramedullary infusion of carrier with/without cPE during the surgery. There were no statistical differences of BMD among the following groups: the cPE (+) treatment group, the cPE (+) MSCs (3 weeks) group (546.0 ± 27.5 mg/mm^3^, *P* > 0.999), and the cPE (+) pMSCs (3 weeks) group (542.1 ± 23.4 mg/mm^3^, *P* > 0.999) (Figure 8).

## Discussion

The current study demonstrated that continuous infusion of contaminated polyethylene particles for 6 weeks increased total number of macrophages, M1 pro-inflammatory macrophages, and the number of osteoclast-like cells positively stained with TRAP and decreased M2 anti-inflammatory macrophages in the region of interest. Local injection of pMSCs acutely during the primary surgery (time 0) or at a delayed time (3 weeks) reversed the decreased proportion of M2 anti-inflammatory macrophages. Unexpectedly, local injection of pMSCs at time 0 resulted in a reduction of BMD at 6 weeks after primary surgery, probably by mitigating the mandatory acute inflammatory reaction. This could also be enhanced by synergism of the pro-inflammatory/osteoblastic effect of both mechanical drilling and intramedullary infusion of carrier with/without cPE during the surgery. The current *in vivo* study highlights the time-dependent effects of pMSCs on bone healing in an inflammatory environment.

An acute, transient inflammatory reaction is necessary for successful bone regeneration and repair; interference with acute inflammation will impair bone healing (Loi et al., 2016a). In the current study, local injection of pMSCs at time 0 resulted in a higher proportion of M2 anti-inflammatory macrophages compared to that of the treatment group with cPE. Thus, local injection of pMSCs at time 0 appeared to interfere with the mandatory acute inflammatory reaction, leading to lower BMD. Using a coculture system with M1 pro-inflammatory macrophages and MC3T3 cells, Loi et al. demonstrated that administration of the anti-inflammatory cytokine Interleukin-4 within 48 hours significantly downregulated oncostatin M (Loi et al., 2016b), which acts through STAT3 to induce osteoblastic differentiation and mineralization (Guihard et al., 2012). Therefore, interventions to facilitate bone healing by modulating acute inflammation should be prudently applied in a temporally sensitive fashion.

Chronic inflammation due to wear particles induced increased pro-inflammatory cytokines and further recruitment of macrophages, leading to subsequent delayed bone healing by the activation of osteoclasts (Goodman, 2007; Goodman et al., 2013, 2019). The current study demonstrated that local injection of pMSCs at 3 weeks (chronic inflammatory phase) polarized macrophages to an M2 anti-inflammatory phenotype, decreased the number of TRAP staining positive cells and increased the positively stained area with ALP. Our previous *in vitro* study also demonstrated that pMSCs increased osteogenic differentiation and polarized macrophages from an M1 pro-inflammatory phenotype to an M2 anti-inflammatory phenotype (Lin et al., 2017). Therefore, local injection of pMSCs during the chronic inflammatory phase may be a potential intervention to mitigate the adverse effects of chronic inflammation of bone.

In the current *in vivo* study, the inhibitory effects of the injection of pMSCs at 3 weeks on cPE-induced chronic inflammation and impaired bone remodeling were found to be comparable with those of injection of MSCs at 3 weeks. This might be related with the later endpoint of this study (total 6 weeks) compared to the previous *in vitro* study with shorter experimental period (24 h) (Lin et al., 2017).

Previous *in vivo* studies using rats used a single inflammatory mediator such as TNF-α (Aktas et al., 2017) and Polyinosinic: polycytidylic acid (polyI:C) (Seather et al., 2016) to precondition MSCs. These approaches shed insight on the mechanisms of Achilles tendon healing (Aktas et al., 2017) and medial collateral ligament healing (Seather et al., 2016). Our previous *in vitro* study showed that preconditioning of MSCs by both LPS and TNF- α enhanced M1 to M2 macrophage polarization and increased osteogenic differentiation of MSCs compared to single or combined treatment of TNF- α and interferon gamma (Lin et al., 2017). Therefore, we adopted a multiple preconditioning strategy using both LPS and TNF- α in the current *in vivo* study.

Several limitations in the present study should be noted. First, the experimental period of the current study (6 weeks) was relatively short-term compared to that of the longer-term biological processes associated with periprosthetic osteolysis in humans. In addition, this study did not consider the effect of the mechanical forces that could also have a protective effect, especially in case of a stable prosthesis. However, this murine *in vivo* model is the first to demonstrate the time-dependent efficacy of MSC-based therapy on bone healing. Second, a single time point of analysis at 6 weeks was conducted in the current study. Several time points of analysis could be even more informative. The current study demonstrated that the bone mineral density significantly decreased, and the number of positively stained cells with TRAP significantly increased in the cPE (+) treatment group at 6 weeks. The current experimental model therefore, appears to simulate chronic inflammatory osteolysis due to wear particles reliably. Third, in the current study, the average size of manufactured polyethylene particles (4.62 ± 3.76 micrometer) is somewhat larger than that in the clinical situation in patients with osteolysis. However, in our previous *in vitro* study, the same manufactured polyethylene particles contaminated by endotoxin resulted in significant pro-inflammatory marker expression (Lin et al., 2017, 2019). Indeed, the current *in vivo* study also showed increased osteoclastogenesis as well as polarization of macrophages to an M1-proinflammatory phenotype due to the same manufactured polyethylene particles contaminated by endotoxin. Thus, the endotoxin-contaminated polyethylene particles were able to initiate inflammatory bone loss. Fourth, in the cPE (−) groups, the Alzet mini-osmotic pumps contained 10% BSA/PBS as carriers in this study. Although these cPE (−) groups did not contain PE particles, the carrier was continuously infused into the intramedullary space; this can be associated with low-grade chronic inflammation. Thus, a non-PE group without carriers should also be considered in the experimental design to address the baseline of biological findings. Several types of non-PE groups have been reported, including saline (Ren et al., 2011; Gibon et al., 2012b), PBS (Gibon et al., 2012a; Nabeshima et al., 2017), serum (Ma et al., 2008), BSA/PBS (Sato et al., 2016; Pajarinen et al., 2017), and insertion of the rod alone (Ma et al., 2008). Ma et al. demonstrated that infusion of UHMWPE particles for 4 weeks reduced the bone volume of the femur by around 10% when compared to the contralateral side of the same mouse in which the rod alone was implanted. However, due to the limited number of animals, we were not able to include non-PE groups without carriers in the current study.

## Conclusion

**FIGURE 9:**
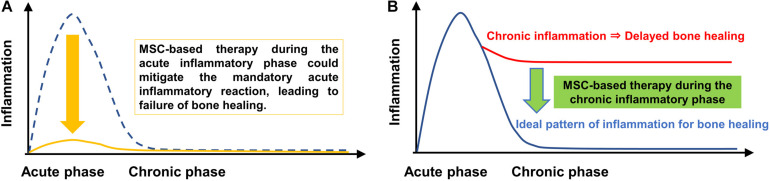
Immunomodulation of bone healing during the early and chronic inflammatory phase. The ideal timeline of inflammation for bone healing consists of an initial and optimal transient stage of acute inflammation followed by the resolution of inflammation, as shown by the blue dotted line in **(A)** and the blue line in **(B)**. MSC-based therapy during the chronic inflammatory phase may be a potential intervention to mitigate the adverse effects of chronic inflammation of bone. However, MSC-based therapy during the acute inflammatory phase could mitigate the mandatory acute inflammatory reaction, leading to suppression of bone healing.

Continuous infusion of polyethylene particles into the femoral canal for 6 weeks induced a prolonged M1 pro-inflammatory macrophage phenotype, a decrease of M2 anti-inflammatory macrophage phenotype, increased osteoclastogenesis and lower BMD. Local injection of MSCs or pMSCs during the chronic inflammatory phase may be a potential intervention to mitigate the adverse effects of contaminated particles on bone. However, local injection of pMSCs during the acute inflammatory phase was found to impair bone healing, probably by mitigating the mandatory acute inflammatory reaction. Thus, the timing of interventions to facilitate bone healing by modulating inflammation should be carefully considered (Figure 9).

## Data Availability Statement

The datasets presented in this article are not readily available because we cannot share the dataset according to rules of our institution. Requests to access the datasets should be directed to TU, takeshiu@stanford.edu.

## Ethics Statement

The animal study was reviewed and approved by Institutional Administration Panel for Laboratory Animal Care at Stanford University (Protocol number: 17566).

## Author Contributions

TU and NZ substantially contributed in research design, or the acquisition, analysis or interpretation of data, drafting the manuscript or revising it critically, and approval of submitted and final versions. TL, YK, and MM substantially contributed in research design, revising the manuscript critically, and approval of submitted and final versions. MU substantially contributed in analysis or interpretation of data, revising the manuscript critically, and approval of submitted and final versions. CR substantially contributed in drafting the manuscript or revising it critically and approval of submitted and final versions. EH substantially contributed in analysis or interpretation of data, revising the manuscript critically, and approval of submitted and final versions. ZY substantially contributed in research design, interpretation of data, revising the manuscript critically, and approval of submitted and final versions. SG substantially contributed in research design, or the acquisition, analysis or interpretation of data, drafting the manuscript or revising it critically, and approval of submitted and final versions. All authors contributed to the article and approved the submitted version.

## Conflict of Interest

The authors declare that the research was conducted in the absence of any commercial or financial relationships that could be construed as a potential conflict of interest.
